# hoodscanR: profiling single-cell neighborhoods in spatial transcriptomics data

**DOI:** 10.1093/bioinformatics/btag609

**Published:** 2026-08-13

**Authors:** Ning Liu, Jarryd Martin, Dharmesh D Bhuva, Jinjin Chen, Mengbo Li, Samuel C Lee, Malvika Kharbanda, Jinming Cheng, Ahmed Mohamed, Arutha Kulasinghe, Yunshun Chen, Chin Wee Tan, Fuyi Li, Jose M Polo, Melissa J Davis

**Affiliations:** South Australian immunoGENomics Cancer Institute (SAiGENCI), Faculty of Health and Medical Sciences, The University of Adelaide, Adelaide, SA 5005, Australia; Bioinformatics Division, The Walter and Eliza Hall Institute of Medical Research, Parkville, Melbourne, Victoria 3052, Australia; Department of Medical Biology, Faculty of Medicine, Dentistry and Health Sciences, University of Melbourne, Parkville, VIC 3010, Australia; Adelaide Centre for Epigenetics, School of Biomedicine, Faculty of Health and Medical Sciences, The University of Adelaide, SA 5005, Australia; Bioinformatics Division, The Walter and Eliza Hall Institute of Medical Research, Parkville, Melbourne, Victoria 3052, Australia; Department of Medical Biology, Faculty of Medicine, Dentistry and Health Sciences, University of Melbourne, Parkville, VIC 3010, Australia; South Australian immunoGENomics Cancer Institute (SAiGENCI), Faculty of Health and Medical Sciences, The University of Adelaide, Adelaide, SA 5005, Australia; Bioinformatics Division, The Walter and Eliza Hall Institute of Medical Research, Parkville, Melbourne, Victoria 3052, Australia; Department of Medical Biology, Faculty of Medicine, Dentistry and Health Sciences, University of Melbourne, Parkville, VIC 3010, Australia; Bioinformatics Division, The Walter and Eliza Hall Institute of Medical Research, Parkville, Melbourne, Victoria 3052, Australia; Department of Medical Biology, Faculty of Medicine, Dentistry and Health Sciences, University of Melbourne, Parkville, VIC 3010, Australia; Bioinformatics Division, The Walter and Eliza Hall Institute of Medical Research, Parkville, Melbourne, Victoria 3052, Australia; Department of Medical Biology, Faculty of Medicine, Dentistry and Health Sciences, University of Melbourne, Parkville, VIC 3010, Australia; Bioinformatics Division, The Walter and Eliza Hall Institute of Medical Research, Parkville, Melbourne, Victoria 3052, Australia; Department of Medical Biology, Faculty of Medicine, Dentistry and Health Sciences, University of Melbourne, Parkville, VIC 3010, Australia; South Australian immunoGENomics Cancer Institute (SAiGENCI), Faculty of Health and Medical Sciences, The University of Adelaide, Adelaide, SA 5005, Australia; Bioinformatics Division, The Walter and Eliza Hall Institute of Medical Research, Parkville, Melbourne, Victoria 3052, Australia; Department of Medical Biology, Faculty of Medicine, Dentistry and Health Sciences, University of Melbourne, Parkville, VIC 3010, Australia; Bioinformatics Division, The Walter and Eliza Hall Institute of Medical Research, Parkville, Melbourne, Victoria 3052, Australia; Department of Medical Biology, Faculty of Medicine, Dentistry and Health Sciences, University of Melbourne, Parkville, VIC 3010, Australia; Frazer Institute, Faculty of Medicine, The University of Queensland, Brisbane, Queensland 4102, Australia; Bioinformatics Division, The Walter and Eliza Hall Institute of Medical Research, Parkville, Melbourne, Victoria 3052, Australia; Department of Medical Biology, Faculty of Medicine, Dentistry and Health Sciences, University of Melbourne, Parkville, VIC 3010, Australia; Bioinformatics Division, The Walter and Eliza Hall Institute of Medical Research, Parkville, Melbourne, Victoria 3052, Australia; Department of Medical Biology, Faculty of Medicine, Dentistry and Health Sciences, University of Melbourne, Parkville, VIC 3010, Australia; Frazer Institute, Faculty of Medicine, The University of Queensland, Brisbane, Queensland 4102, Australia; South Australian immunoGENomics Cancer Institute (SAiGENCI), Faculty of Health and Medical Sciences, The University of Adelaide, Adelaide, SA 5005, Australia; South Australian immunoGENomics Cancer Institute (SAiGENCI), Faculty of Health and Medical Sciences, The University of Adelaide, Adelaide, SA 5005, Australia; Adelaide Centre for Epigenetics, School of Biomedicine, Faculty of Health and Medical Sciences, The University of Adelaide, SA 5005, Australia; Department of Anatomy and Developmental Biology, Monash University, Clayton, VIC 3800, Australia; South Australian immunoGENomics Cancer Institute (SAiGENCI), Faculty of Health and Medical Sciences, The University of Adelaide, Adelaide, SA 5005, Australia; Bioinformatics Division, The Walter and Eliza Hall Institute of Medical Research, Parkville, Melbourne, Victoria 3052, Australia; Department of Medical Biology, Faculty of Medicine, Dentistry and Health Sciences, University of Melbourne, Parkville, VIC 3010, Australia; Computational Biology, Isomorphic Labs, Bishopsgate, London, EC2M 4RB, United Kingdom

## Abstract

**Motivation:**

Understanding complex cellular neighborhoods has provided new insights into tissue biology. Accurate neighborhood identification is crucial, yet existing methods often focus on hard assignment and do not generate cell-specific neighborhood profiles at single-cell level.

**Results:**

We developed hoodscanR, a Bioconductor package identifying and analyzing cellular neighborhoods in spatial data. The central output of hoodscanR is a cell-level neighborhood probability profile, which represents partial membership of each cell across multiple annotation-defined neighborhoods. This probabilistic representation supports downstream analyses including neighborhood visualization, uncertainty assessment, neighborhood-based clustering and neighborhood-aware differential expression. Applying hoodscanR to breast and lung cancer datasets, we showcase its ability to characterize mixed tissue environments and identify transcriptional changes in tumor cells from distinct spatial neighborhoods.

**Availability:**

The hoodscanR package is publicly available in Bioconductor at https://bioconductor.org/packages/release/bioc/html/hoodscanR.html.

## Graphical Abstract

**Graphical Abstract:**
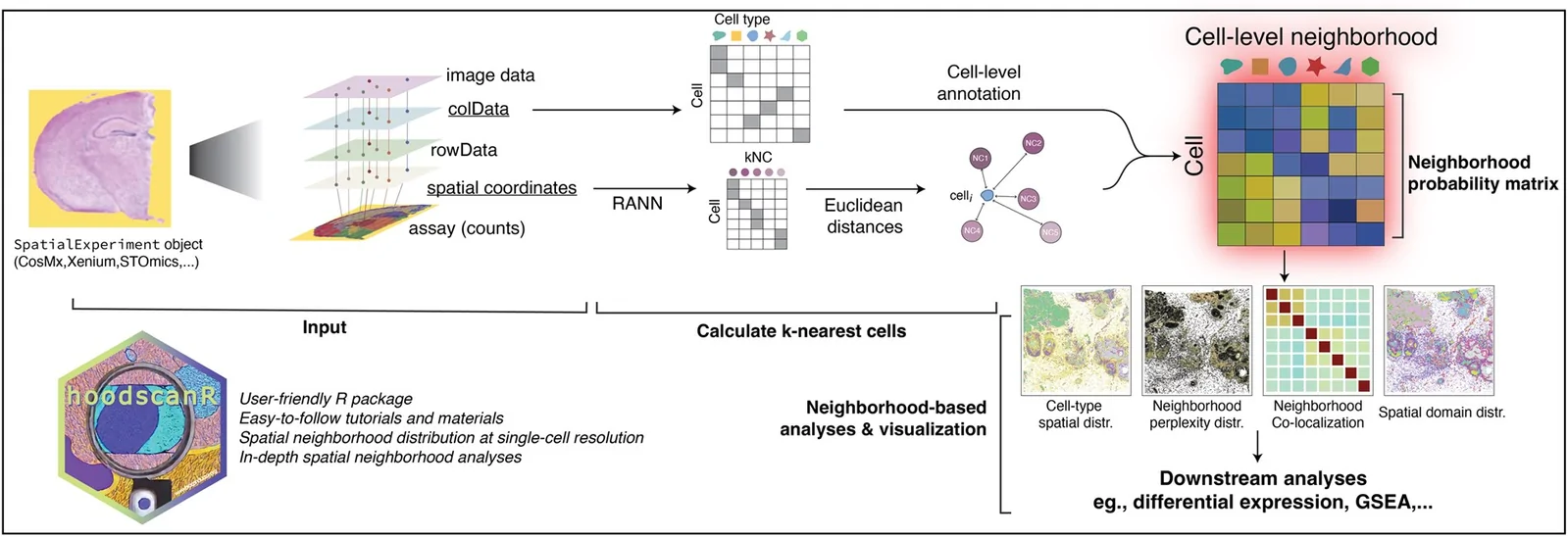

## 1 Introduction

Spatial transcriptomics stands out as a powerful technology, offering spatial perspective that goes beyond bulk RNA-seq and single-cell RNA-seq. However, many analyses resembling scRNA-seq-like approaches, often disregarding the spatial context of the data, failing to harness the cell coordinates. Thus, the lack of appropriate methods has created a pressing demand for innovative analytical tools capable of fully exploiting these datasets. Cellular neighborhood analysis, a powerful way to fully utilize cell spatial information, becomes particularly important when applied to single-cell level spatial transcriptomics data. Bioinformatics tools are needed to identify and characterize the spatial microenvironments or neighborhoods in which cells reside, as these regions may harbor crucial tissue micro-environment (TME) biology that influences the fundamental tissue biology, physiology as well as responses to therapy and disease progression. Therefore, understanding these neighborhoods is key for the full utilization of the spatial data and to provide researchers with novel insights into cellular interactions and communications within the TME, offering a nuanced understanding of the complex biological processes at play. Such insights hold the potential to enhance our understanding of complex diseases like cancer and contribute to the development of more effective therapies.

In recent years, there has been a growing trend in the development of methods dedicated to conducting neighborhood analyses. These methods range from clustering-based approaches (He *et al*. 2022) to graph network-based strategy (Warchol *et al*. 2023). Widely used toolkits Squidpy (Palla *et al*. 2022) and Giotto (Dries *et al*. 2021) allow neighborhood analyses via enrichment tests using a graph-based approaches. Additionally, many tools have been developed to detect spatial domains, including BuildNicheAssay from Seurat (Hao *et al*. 2024), MERINGUE (Miller *et al*. 2021), BANKSY (Singhal *et al*. 2024), BayesSpace (Zhao *et al*. 2021), STAGATE (Dong and Zhang 2022), SpaGCN (Hu *et al*. 2021) and UTAG (Kim *et al*. 2022). Nevertheless, despite these advancements, there are critical gaps in existing methodologies. Most notably, while some existing tools can detect spatial domains that comprise multiple cell types, they do not provide partial membership at a single-cell level. Furthermore, current tools lack the capability to provide cell-level neighborhood annotations. This critical feature is essential for a comprehensive characterization of the spatial context surrounding each cell. To address these limits, we developed hoodscanR, a Bioconductor R package designed to perform comprehensive neighborhood analyses on spatial transcriptomics data. Unlike methods that mainly assign cells to a single spatial domain, hoodscanR generates cell-level neighborhood probability profiles that capture partial membership across multiple neighborhoods. These profiles provide a quantitative representation of the spatial context surrounding each cell and can be used for downstream visualization, uncertainty assessment, co-localization analysis, neighborhood-based clustering and neighborhood-based differential expression analysis. Notably, in this study, we use the term “neighborhood” to refer to the local cellular environment surrounding an individual cell. In contrast, we use “spatial domain” to refer to groups of cells with similar neighborhood profiles, which can be derived through downstream clustering rather than being the primary output of the method.

## 2 Methods

### 2.1 Cell type annotation

Cell type annotation for the NSCLC data was carried out with modifications as previously described in Tan *et al*. 2024 (Tan *et al*. 2024). Briefly, specific modifications include using SCTransform from the Seurat package (Hao *et al*. 2021) to normalize filtered counts from the quality control step. Cell type annotation for the NSCLC data was generated using InSituType (Danaher *et al*. 2022), with the Single-cell Lung Cancer Atlas (LuCA) (Salcher *et al*. 2022) as the reference. For Xenium data, cell type annotation was obtained from the Janesick *et al.* paper (Janesick *et al*. 2023).

### 2.2 Metrics calculation for neighborhood probability distribution

Perplexity serves as a fundamental metric for summarizing the neighborhood probability distribution. The perplexity P(x) for a given cell x is calculated as:


$$P(x)=2^{H(x)}$$

where H(x) represents the Shannon entropy (Shannon 1997) of the neighborhood probability distribution of cell x, defined as:


$$H(x)=-\sum_{i=1}^{n}p(x_{i})\log_{2}p(x_{i})$$

where $p(x_{i})$ is the probability of cell *x* located in the *i*-th neighborhood and *n* is the total number of distinct neighborhoods. In hoodscanR, perplexity can be calculated using the calcMetrics function. To assess the statistical significance of the observed perplexity values within cell neighborhoods, we employed an empirical permutation test. For each neighborhood, we generated a distribution of perplexity values by randomly shuffling the spatial coordinates of cells and recalculating the perplexity across 1000 permutations. The empirical *P*-value for each neighborhood was then calculated as the proportion of permuted perplexity values that were greater than or equal to the observed perplexity value, adjusted for the finite number of permutations:


$$P_{\mathrm{empirical}}=\frac{\sum_{i=1}^{N}1(P_{\mathrm{obs}}\geq P_{i})+1}{N+1}$$

where $P_{\mathrm{obs}}$ is the observed perplexity for a given neighborhood, $P_{i}$ represents the perplexity from the *i*-th permutation, 1(.) is an indicator function that equals 1 when the condition inside is true and 0 otherwise, and N is the total number of permutations. This correction ensures that the empirical *P*-values are properly calibrated, even with a limited number of permutations, thus providing a robust measure of statistical significance.

### 2.3 Hyperparameter *k* and *τ* testing

To test the effect different parameter *k* values have on the results of hoodscanR, we first test a range of *k* values (10, 50, 100, 200, 500, 1000), using the default *τ* setting from the scanHoods function. We then computed the Pearson correlation between the resulting probability matrices to assess consistency of the outcomes across different k. For testing the *τ* parameter, we fixed *k* = 100 and examined a range of *τ* values, which were derived from different scaling of the distance matrix (see Availability of data and materials). In practice, the default parameters provide a useful starting point for most datasets. Smaller *k* and *τ* are suitable when the aim is to capture highly local profile, while larger *k* and *τ* are useful when broader structures are of interest.

### 2.4 Benchmarking spatial domain identification

To benchmark hoodscanR against other state-of-the-art methods in identifying domains, we selected 12 publicly available datasets. Due to the large size of the breast cancer datasets, some tools were unable to process these datasets. We therefore downsampled these slides to 10 000 and 50 000 cells, while maintaining the original cell type proportion distributions. Annotated region labels or pathological annotations in these datasets were used as the ground truth for spatial domains. For each method, we calculated a composite performance score to assess the accuracy of spatial domain identification. This score was computed as the mean of four metrics: Adjusted Rand Index (ARI), Normalized Mutual Information (NMI), purity, and homogeneity: Score = (ARI + NMI + purity + homogeneity)/4. ARI measures the similarity between the predicted and true clusters, adjusting for chance. NMI quantifies the amount of information shared between the predicted and true clusters. ARI and NMI here are calculated using the aricode R package. Purity measures the extent to which each cluster contains only members of a single class. Homogeneity ensures that all the clusters contain only data points which are members of a single class. Purity and Homogeneity here are calculated using sklearn library. Additionally, a time penalty was applied: the process was terminated if a method failed to complete the processing within 24 hours.

### 2.5 Unsupervised clustering of neighborhood distribution

To perform unsupervised clustering of the neighborhood distribution, the *K*-means clustering algorithm was used. To determine the optimal number of clusters (*k*) for the *K*-means clustering algorithm, we used the elbow method. In a nutshell, we first calculated the within-cluster sum of squares for a range of *k* values and then identified the point at which the distortion or inertia starts decreasing in a linear fashion.

### 2.6 Differential expression analysis

To conduct the differential expression (DE) analysis on the NSCLC dataset and identify DE genes, we obtain pseudo-bulk samples using the summarizeAssayByGroup function from the scuttle R package (McCarthy *et al*. 2017). Subsequently, the standR package (Liu *et al*. 2024) was used to assess relative log expression (RLE) and perform principal component analysis (PCA). Following this, the limma-voom pipeline (Law *et al*. 2014) was used, incorporating slide information as a covariate in the linear model. Multiple testing adjustment using the Benjamini–Hochberg procedure was then applied to identify DE genes (FDR < 0.05).

### 2.7 Gene-set enrichment analysis and visualization

Gene-sets from the Molecular Signatures Database (MsigDB, v7.2), including Hallmarks, C2 and C5, along with KEGG gene-sets, were obtained. Gene-set enrichment analysis (GSEA) was performed using fry from the limma package (v3.58.1). The results of GSEA were visualized using vissE (Bhuva *et al*. 2024).

### 2.8 Data availability

The Nanostring CosMx non-small cell lung cancer (NSCLC) data in this study was sourced from the Nanostring website: https://nanostring.com/products/cosmx-spatial-molecular-imager/ffpe-dataset/nsclc-ffpe-dataset/. The 10X Xenium breast cancer datasets here were retrieved from the 10X publicly available database at https://www.10xgenomics.com/datasets. The MERFISH mouse colon data was downloaded from https://doi.org/10.5061/dryad.rjdfn2zh3. The STARmap mouse cortex data was sourced from the Wang *et al.* study (Wang *et al*. 2018).

### 2.9 Code availability

The code used for performing the described analyses is available in GitHub at https://github.com/ningbioinfo/hoodscanR_manuscript_code and in Zenodo (10.5281/zenodo.20540938). hoodscanR package is available in Bioconductor at https://bioconductor.org/packages/release/bioc/html/hoodscanR.html.

## 3 Results

**Figure 1:**
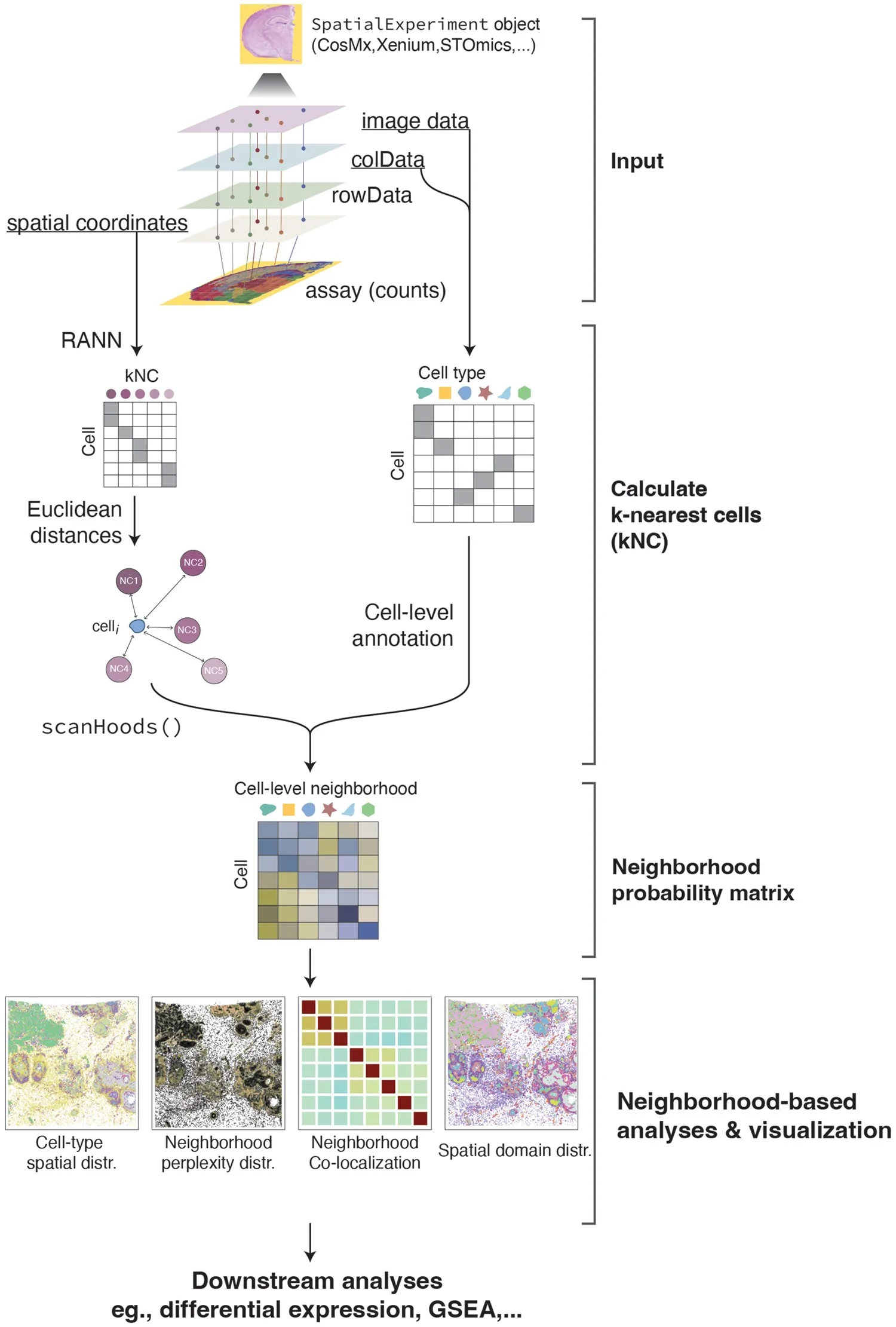
Computational workflow of hoodscanR. hoodscanR takes a SpatialExperiment object containing spatial coordinates, expression data and cell-level annotations as input. The package identifies the k-nearest neighboring cells based on spatial proximity and calculates distances between each cell and its local neighbors. These distances are combined with user-defined cell annotations to generate the central output of hoodscanR: a cell-level neighborhood probability matrix. Each row of this matrix represents a cell, and each column represents the probability that the cell resides within a given neighborhood type. This matrix supports downstream visualizations, perplexity and uncertainty assessment, co-localization analysis, neighborhood-based clustering and differential expression analysis.

hoodscanR uses an efficient pipeline to investigate spatial neighborhood relationships among cells (Fig. 1), it uses the SpatialExperiment infrastructure as the backbone of the analysis, significantly increases the compatibility of intermediate results from hoodscanR with diverse Bioconductor packages tailored for various analyses. At the core of hoodscanR, the nearest cells are searched using the Approximate Nearest Neighbor (ANN) search algorithm (Arya *et al*. 1998). This process outputs a list of indices representing the nearest neighbors of each cell, denoted as: $\mathrm{ANN}(x)=\{x_{1},x_{2},\ldots ,x_{k}\}$. hoodscanR then calculates the Euclidean distance between each cell and its k-nearest neighbors. This results in a distance matrix D, where each element $D_{ij}$ represents the distance between cell $x_{i}$ and its neighbor $x_{j}$ from ANN(x) (Fig. 1). Simultaneously, cell-level annotations provided by users, such as cell types, are used to construct a cell annotation matrix A, which describes the organization of cells based on their distances to neighboring cells. Each entry A indicates whether cell $x_{i}$ belongs to annotation group j:


$$A=\{\begin{array}{cc} 1 & if\;\mathrm{cell}\;x_{i}\in \{x_{1},x_{2},\ldots ,x_{k}\}\;\mathrm{belongs}\;to\;\mathrm{annotation}\;\mathrm{group}\;j\\ 0 & o\mathrm{therwise} \end{array}$$

The fundamental function of hoodscanR is to identify cellular neighborhoods. It achieves this by using a modified SoftMax function with a hyperparameter *τ* (tau), which governs the shape of the resulting probability distribution and provides control over the influence of neighboring cells. The algorithm is expressed as follows:


$$p_{h_{j}}(x;\tau )=\frac{\sum_{i=1}^{k}1_{h_{j}}(x_{i})\cdot \;\text{exp}\;\{-\frac{d^{2}(x,x_{i})}{\tau }\}}{\sum_{i=1}^{k}\;\text{exp}\;\{-\frac{d^{2}(x,x_{i})}{\tau }\}},\;x,x_{i}\in \{x_{1},x_{2},\ldots ,x_{k}\}$$

$$1_{h_{j}}(x_{i})=\{\begin{array}{cc} 1 & if\;A[i,j]\;is\;1\\ 0 & o\mathrm{therwise} \end{array}$$

where: $p_{h_{j}}(x;\tau )$ denotes the probability of cell x residing within the local neighborhood $h_{j}.\;d(x,x_{i})\;$signifies the spatial Euclidean distance between cell x and its neighboring cell $x_{i}.\;\tau$ stands as the hyperparameter, facilitating fine-tuned modulation of the impact of neighboring cells. $h_{j}$ denotes the cell neighborhood a, defined by the cell-level annotations provided by users. For example, if cell types were provided, $h_{j}$ means cell type j neighborhood. 1(.) is the indicator function, which checks whether cell $x_{i}$ belongs to the neighborhood $h_{j}$ as per the annotation matrix A.

Upon the aggregation of probabilities by user-defined annotation, such as cell types, hoodscanR generates a probability matrix P, where each value represents the probability of each cell belonging to a specific neighborhood (Fig. 1). This matrix is the central output of hoodscanR, capturing partial neighborhood membership at single-cell resolution and forms the basis for downstream analyses.

To investigate how the hyperparameter *k* and *τ* affects the results generated by hoodscanR, we conducted an examination of the matrix across a range of *k* and *τ* values (see Methods). This revealed that different *k* values generate highly similar results, with a mean Pearson correlation coefficient of 0.93 (Fig. S1, available as supplementary data at *Bioinformatics* online). Regarding *τ*, smaller values assign greater weights to nearby cells, while larger *τ* values consider more distant cells as contributors to the neighborhood (Fig. S2, available as supplementary data at *Bioinformatics* online). Thus, the choice of *τ* becomes essentially linked to the specific biological questions being addressed. For example, smaller *τ* values are more suitable for analyses focused on local interactions, while larger *τ* values are ideal for capturing more global spatial relationships. Together, these results suggest that hoodscanR is robust across a broad range of *k* values, while *τ* provides an interpretable way to tune the spatial scale of neighborhood detection. For most applications, we recommend starting with the default settings and then adjusting *k* and *τ* according to the biological question. Smaller *k* or *τ* values are preferable for identifying local cell-cell interactions, sharp boundaries or immediate microenvironmental effects. Larger *k* or *τ* values are more appropriate for capturing broader tissue architecture or spatial domains that extend beyond immediate neighbors.

After neighborhood identification, hoodscanR offers a diverse suite of downstream neighborhood analysis tools (Fig. 1). Users can apply these tools to visualize spatial relationships, evaluate co-localization patterns, perform spatial neighborhood clustering of cells, and obtain cell-level neighborhood annotations. In conclusion, hoodscanR provides a powerful and flexible method for spatial neighborhood identification and analyses.

### 3.1 Benchmarking hoodscanR in spatial domains identification

**Figure 2:**
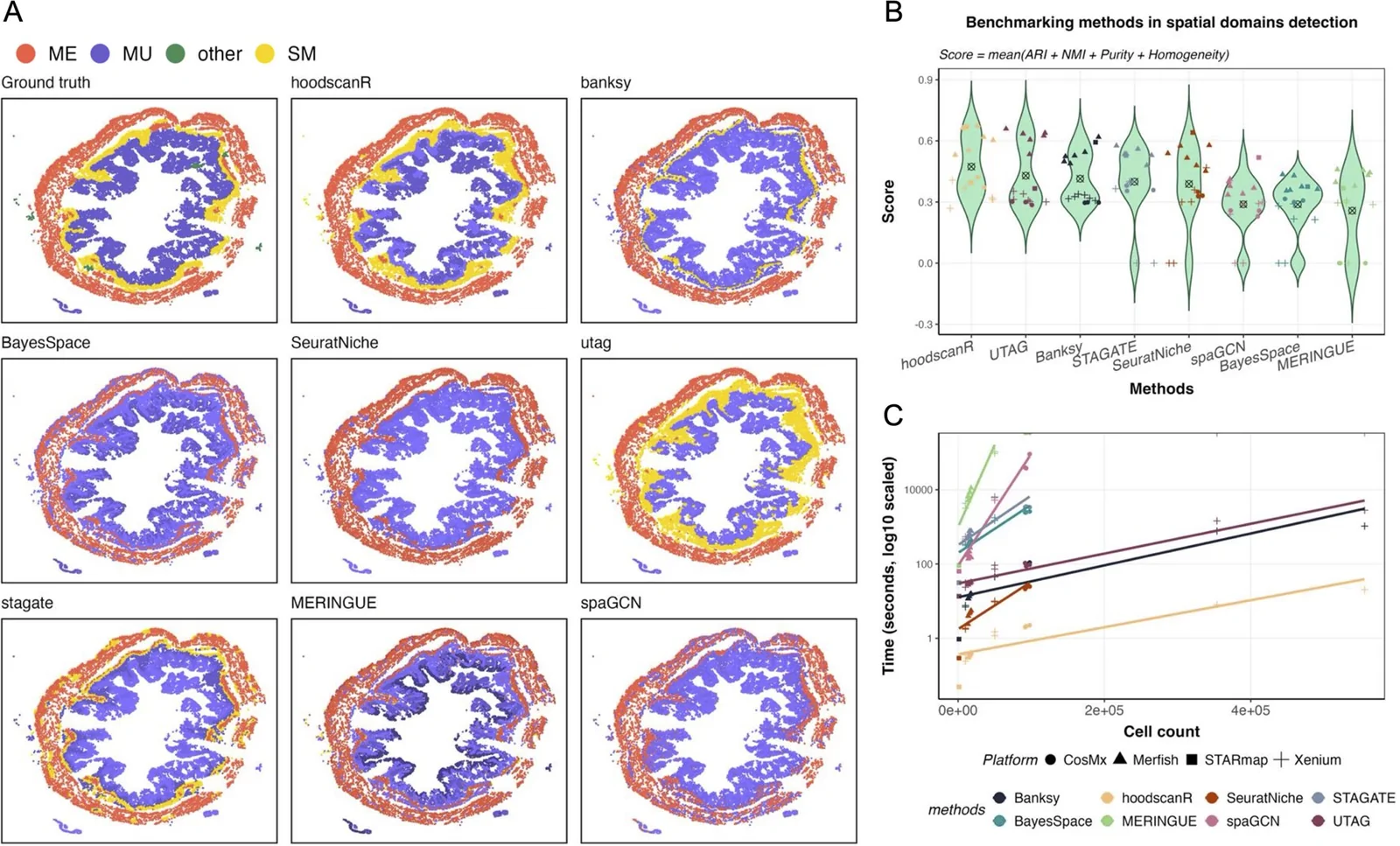
Benchmarking hoodscanR against other methods in detecting spatial domains. (A) Spatial maps of the MERFISH mouse colon data, colored by the spatial domains detected from different methods compared to the ground truth domain annotations, including muscularis externa (ME), mucosa (MU), submucosa (SM) and other (top left corner). (B) Violin plots showing the performance score of each method across all tested datasets, ordered from the highest to the lowest mean score. (C) Computational efficiency of each method, plotted as the log10-scaled time (in seconds) required to process datasets. Shapes represent the platform of the dataset, colors denote the methods, and the lines are generalized linear smooths indicating overall trends for each method.

hoodscanR allows users to perform unsupervised clustering, enabling the classification of cells based on their spatial relationships, identifying neighborhood-driven spatial domains. To evaluate the effectiveness and robustness of the neighborhood-based spatial domain found in hoodscanR, we conducted a benchmarking experiment against several state-of-the-art methods (see Methods). This benchmark experiment involved detecting spatial domains of 16 publicly available datasets, including NSCLC (He *et al*. 2022), mouse colon (Cadinu *et al*. 2024), mouse cortex (Wang *et al*. 2018) and breast cancer (Janesick *et al*. 2023, Bhuva *et al*. 2024). The datasets were chosen because the tissue is well-annotated with region labels or they have pathological annotation that can be served as ground truth. hoodscanR was benchmarked against 7 other methods: BuildNicheAssay from Seurat, Banksy, BayesSpace, MERINGUE, SpaGCN, Stagate and Utag (Fig. 2A and Figs S3–S7, available as supplementary data at *Bioinformatics* online). In these experiments, hoodscanR outperformed other methods, achieving the highest average performance across all tested datasets (Fig. 2B). Moreover, hoodscanR showed strong computing efficiency compared with other methods (Fig. 2C). This efficiency reflects the use of approximate nearest neighbor search, and the reduction of local spatial information into a compact cell-by-neighborhood probability matrix. Additionally, hoodscanR can recapitulate tissue architecture in a biologically coherent manner. For example, in the MERFISH mouse colon dataset (Fig. 2A), hoodscanR accurately delineates four concentric layers, mucosa (MU), submucosa (SM), muscularis externa (ME), and other, all of which contain multiple cell types. This result closely mirrors the ground truth. Taken together, these results highlight strengths of hoodscanR in domain identification across large-scale spatial transcriptomics datasets. To further evaluate the robustness of hoodscanR across different resolutions of annotations, we conducted an experiment using high-, medium- and low-resolution annotations as inputs. The high-resolution annotations included detailed cell types, such as CD4+ T cells. The medium-resolution annotations combined all T cells into a single category, and the low-resolution annotations further grouped all immune cells together. Despite the reduction in resolution, the identified domains have an NMI greater than 0.8 (Fig. S8, available as supplementary data at *Bioinformatics* online). Taken together, these results showcase the power of hoodscanR in accurately identifying neighborhood-based spatial domains in a scalable and efficient manner.

### 3.2 hoodscanR identifies cellular neighborhoods in cancer

**Figure 3:**
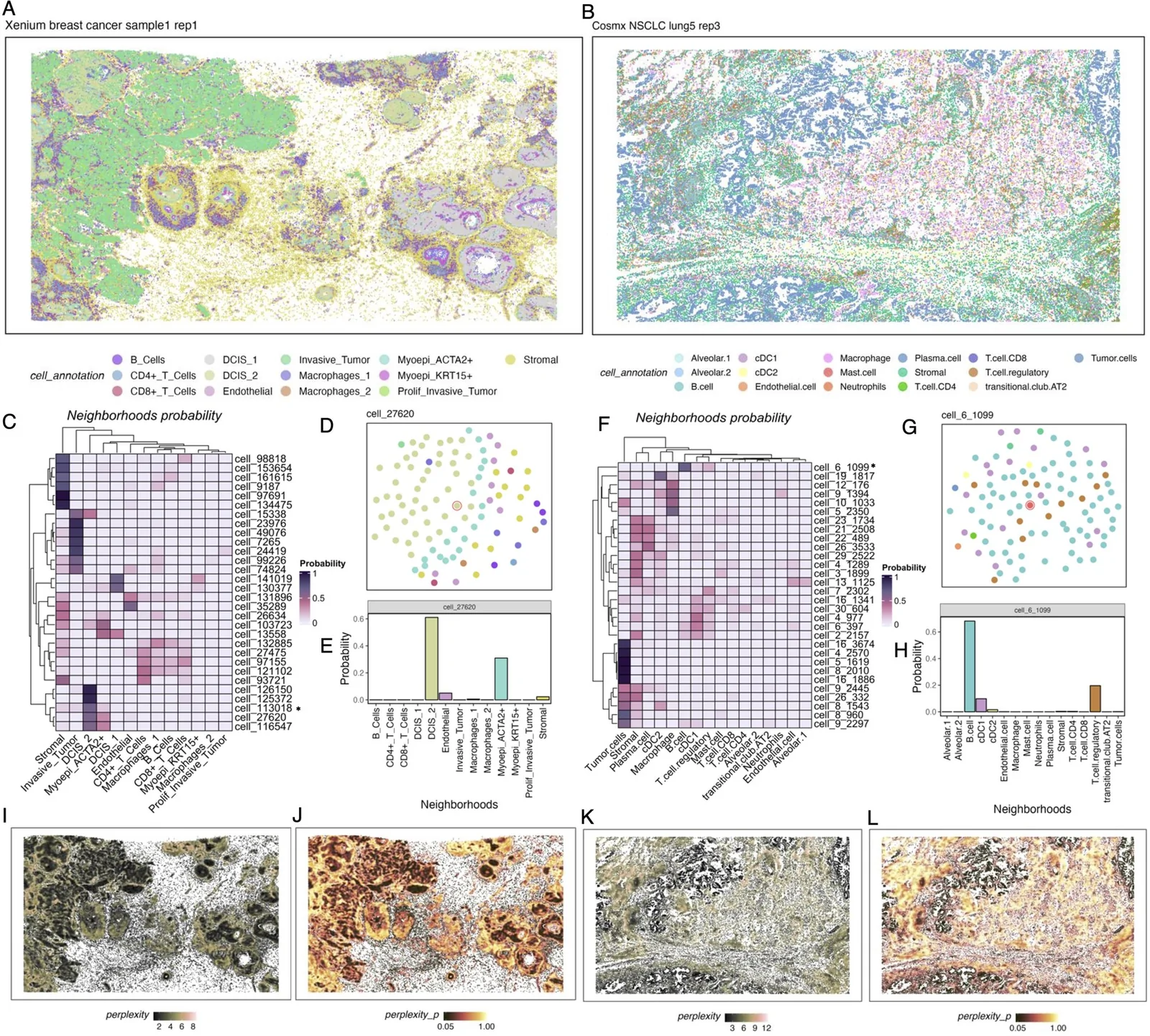
Neighborhood identification in 10X Xenium breast cancer data and Nanostring CosMx NSCLC data. Cell type spatial distribution in the breast cancer data (A) and NSCLC data (B). Neighborhood distribution visualization via heatmap of randomly selected 30 cells from breast cancer data (C) and NSCLC data (F), darker color means higher probability of the cell located in specific cell type neighborhood. The cell type spatial distribution in the spatial area around the selected cells (marked by * in the heatmap) in breast cancer data (D & E) and NSCLC data (G & H). Perplexity spatial distribution of cells in the breast cancer data (I) and NSCLC data (K). *P*-value distribution of perplexity in the breast cancer data (J) and NSCLC data (L).

We performed neighborhood identification on two publicly available breast cancer (Fig. 3A) and non-small cell lung cancer (NSCLC) (Fig. 3B) spatial transcriptomics datasets to demonstrate hoodscanR applicability. hoodscanR allows us to perform neighborhood identification by profiling neighborhood distributions, representing the probability of a cell being situated within each distinct cell-type neighborhood (Fig. 3C and F).

To validate the accuracy of hoodscanR in characterizing these cellular neighborhoods, we focus on one randomly selected cell for each dataset by exploring the distribution of cell types within their spatial area (Fig. 3D, E, G, and H). For instance, we examined a ductal carcinoma *in situ* (DCIS) grade 2 cell, an early form of breast cancer cells, from the Xenium data (Fig. 3C and D: cell ID 27620), where we observed 47 DCIS grade 2 cells and 21 ACTA2+ myoepithelial cells from the nearest 100 neighboring cells (Fig. 3D). Consistently, hoodscanR assigned probabilities of 61.02% for residing in the DCIS grade 2 neighborhood and 30.96% for the ACTA2+ myoepithelial neighborhood for this specific cell (Fig. 3E). Similarly, when assessing a stromal cell within the NSCLC data (Fig. 3F–H). These examples demonstrate the power of hoodscanR in accurately characterizing cellular neighborhoods.

Furthermore, hoodscanR introduces an additional analytical dimension by enabling the computation of uncertainty, which is measured by perplexity, and performing permutation test for each cell (see Methods). Perplexity provides overall measurement of the uncertainty and complexity of cell neighborhoods (Fig. 3I and K). This in turn reveals regions of the TME with distinct cellular compositions and areas with complex interactions between cell types. *P*-values of perplexity (Fig. 3J and L) can be obtained via an empirical permutation test (see Methods). This allows users to identify regions with significantly higher perplexity from tissue. Altogether, these metrics provide an understanding of the heterogeneity and complexity present within tissues, allowing researchers to gain novel insights and make discoveries in the spatial transcriptomics landscape.

### 3.3 hoodscanR detects changes between tumor cells from different neighborhoods

**Figure 4:**
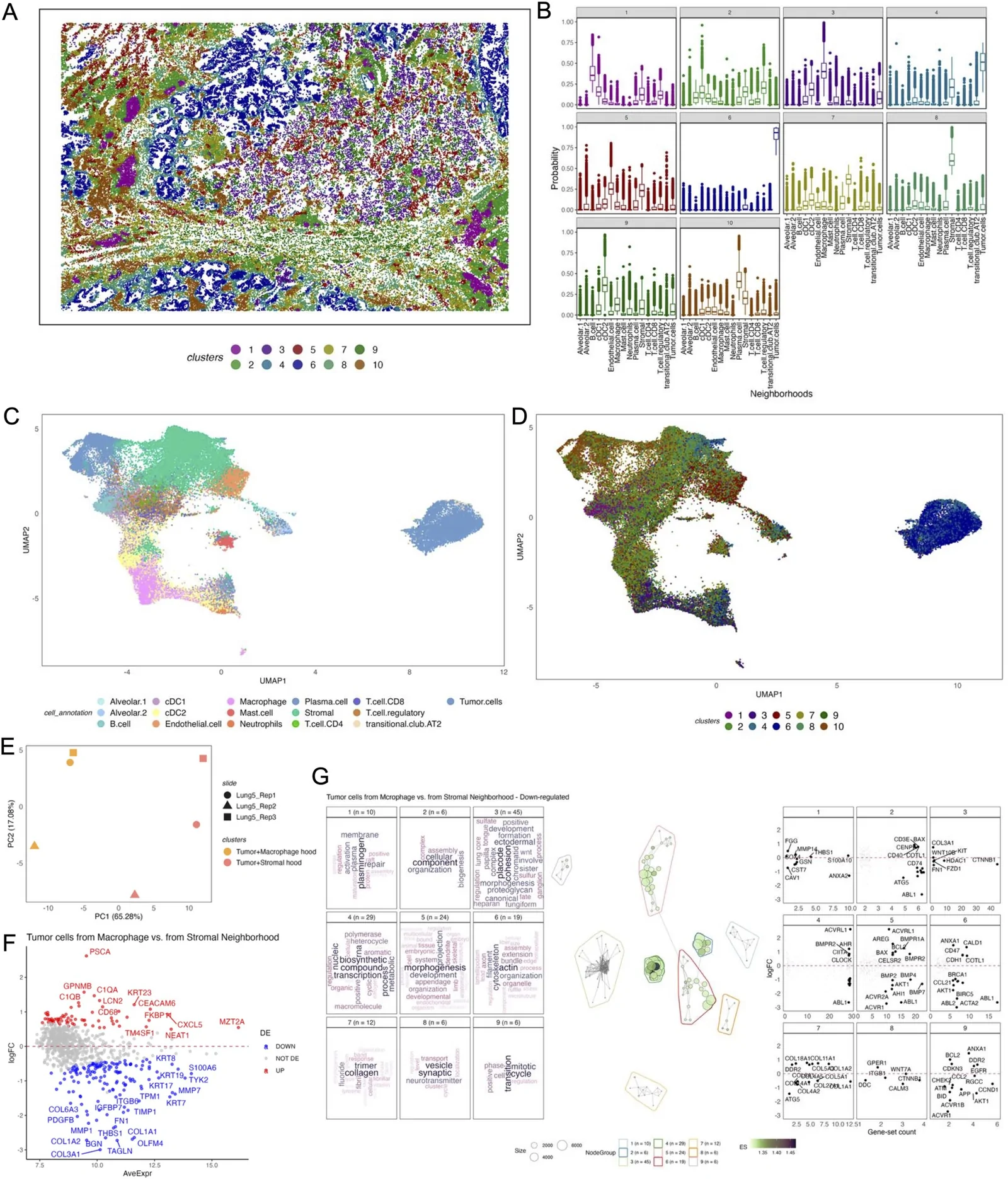
Neighborhood-based transcriptomics analysis in CosMx NSCLC data. (A) Spatial location plot of the slide with colors stratified by neighborhood-based clusters. (B) The neighborhood distribution profiles of each neighborhood-based cluster identified in the tissue slide. (C) UMAP of expression of cells in the data, colors denote cell types. (D) UMAP of expression of cells in the data, colors denote the identified neighborhood-based clusters. (E): PCA of pseudo-bulk samples of tumor cells from two different neighborhood clusters across three consecutive slides. Colors denote clusters and shapes denote replicates. (F) MA plot describing the outcome of the DE analysis. Colors indicate up- (red) or down-regulated (blue) genes. (G) vissE visualization of significantly enriched gene-sets from the down-regulated DE genes in the comparison between tumor cells from macrophage neighborhood and tumor cells from stromal neighborhood. Left panel are word cloud plots describing gene-set clusters of different biological themes, middle panel is the gene-set overlap network graph of gene-sets, and right panel is the fold-change (log2-scaled) for genes belonging to each gene-set cluster.

In the NSCLC data, we further applied clustering to delineate 10 distinct clusters (see Methods), each representing a unique spatial pattern within the tissue (Fig. 4A), demonstrating complex spatial associations. For example, cluster 1, a candidate cluster for TLS, corresponds to a neighborhood including B cells, cDC1 cells, and stromal cells (Fig. 4B–1), cluster 3 aligns with macrophages and cDC2 cells (Fig. 4B–3), and cluster 6 corresponds to tumor cells (Fig. 4B–6). We then used uniform manifold approximation and projection (UMAP) to visualize the expression data. This facilitates the visualization of cell lineage (Fig. 4C) alongside identified neighborhood-based clusters (Fig. 4D). A distinctive feature observed is the dispersion of cells of the same cell type across different neighborhood clusters, signifying diverse spatial neighborhood profiles. For example, a substantial proportion (76.14%) of macrophages (pink points in Fig. 4C) are distributed across various neighborhood clusters, including cluster 3 (43.63%), indicative of the macrophage + cDC2 + tumor neighborhood, clusters 7 (12.31%), and cluster 9 (20.2%), representing the stromal + cDC2 neighborhood.

hoodscanR can study the relationship between spatial neighborhoods and transcriptional changes. To demonstrate this, we conducted a nuanced analysis by extracting tumor cells from two distinct neighborhood clusters: the stromal cluster and the macrophage cluster (Fig. S9, available as supplementary data at *Bioinformatics* online). Interestingly, different spatial neighborhoods contribute substantially to the variation along the first principal component from a principal component analysis (PCA) (Fig. 4E). Subsequently, we performed a differential expression (DE) analysis using the limma-voom pipeline (Law *et al*. 2014), identifying 220 DE genes from 832 genes, including 73 up-regulated and 147 down-regulated genes when comparing tumor cells from the macrophage neighborhood to those from the stromal neighborhood (Fig. 4F and Supplementary file 1, available as supplementary data at *Bioinformatics* online).

Additionally, we performed gene-set enrichment analysis on the DE genes with MSigDB gene-sets (Subramanian *et al*. 2005, Liberzon *et al*. 2011), detecting 384 significantly enriched gene-sets (Supplementary file 2, available as supplementary data at *Bioinformatics* online). We further performed clustering on gene-sets using vissE (Bhuva *et al*. 2024), identifying gene-sets networks (Fig. 4G and Fig. S10, available as supplementary data at *Bioinformatics* online, middle panel). Notably, we observe pathways enriched in down-regulated DE genes related to collagen, such as collagen-activated tyrosine kinase receptor signalling pathway and collagen metabolic and catabolic process (Fig. 4G). These pathways are accompanied by the differential expression of key collagen-related genes, such as COL1A1, COL11A1 and COL5A1 (Fig. 4E right panel). Previous studies have found that the overexpression of collagen genes such as COL11A1 (Shen *et al*. 2016) and COL3A1 (Wang *et al*. 2022) in NSCLC may indicate poor prognosis and drug resistance, and COL1A1 is correlated with immune infiltration in NSCLC (Geng *et al*. 2021). Our finding of these genes that are expressed significantly more in the tumor cells from the macrophage neighborhood than tumor cells from the stromal neighborhood can potentially lead to a more detailed investigation of the biological mechanism about transcriptional changes within the context of cancer spatial TME studies. In essence, hoodscanR introduces a novel perspective in spatial transcriptomics, allowing the identification of transcriptomic changes across subtly different spatial neighborhoods and providing insights into the spatial organization within the TME.

## 4 Discussion

Spatial technologies are pushing the limits toward profiling spatial transcriptomics at single-cell level. To make the best use of these cutting-edge technologies, we developed hoodscanR, a powerful Bioconductor package for revealing spatial cellular relationships via spatial cellular neighborhoods identification and neighborhood-based downstream analyses. hoodscanR is a cell-based method, emphasizing the critical role of accurate cell segmentation, which depends upon upstream image processing and cell segmentation methods. The segmentation process, which determines how individual cells are identified and their spatial coordinates are established, is fundamental to the accurate detection of cellular neighborhoods.

The significance of hoodscanR in identifying and analyzing neighborhoods becomes particularly crucial in the context of complex diseases, notably in cancer research. Neighborhood information is indispensable for unraveling complex disease etiology, especially so for understanding disease progression. In cancer research, where the TME plays an important role in dictating therapy responses, the ability offered by hoodscanR to identify neighborhoods offers a unique perspective to investigate novel mechanisms underlying the transition of cancer cells at both transcriptomic and proteomic level. Notably, our findings from the NSCLC dataset identified cellular neighborhoods that are potentially associated with the presence of TLS, which correlated with positive clinical results (Ren *et al*. 2022). This capability offers the potential to contribute valuable insights into the progression of cancer, paving the way for the development of novel therapeutic strategies.

## Supplementary Material

btag609_Supplementary_Data
