# Supplementary material for: hoodscanR: profiling single-cell neighborhoods in spatial transcriptomics data
Source: Bioinformatics. 2026 Aug 13;42(8):btag609. doi: 10.1093/bioinformatics/btag609 (PMC13503035; doi:10.1093/bioinformatics/btag609)
Supplement: Supplementary file 1 [file btag609_supplementary_data.zip › SupplementaryData.docx]

Supplementary Data for

**hoodscanR: profiling single-cell neighborhoods in spatial transcriptomics data**

Ning Liu^1,2,3,4,*^, Jarryd Martin^2,3^, Dharmesh D Bhuva^1,2,3^, Jinjin Chen^2,3^, Mengbo Li^2,3^, Samuel C. Lee^2,3^, Malvika Kharbanda^1^, Jinming Cheng^2,3^, Ahmed Mohamed^2,3^, Arutha Kulasinghe^5^, Yunshun Chen^2,3^, Chin Wee Tan^2,3,5^, Fuyi Li^1^, Jose M Polo^1,4,6,*^, Melissa J Davis^1,2,3,7,*^

^1^ South Australian immunoGENomics Cancer Institute (SAiGENCI), Faculty of Health and Medical Sciences, The University of Adelaide, Adelaide, SA 5005, Australia

^2^ Bioinformatics Division, The Walter and Eliza Hall Institute of Medical Research, Parkville, Melbourne, Victoria 3052, Australia

^3^ Department of Medical Biology, Faculty of Medicine, Dentistry and Health Sciences, University of Melbourne, Parkville, VIC 3010, Australia.

^4^ Adelaide Centre for Epigenetics, School of Biomedicine, Faculty of Health and Medical Sciences, The University of Adelaide, SA 5005, Australia

^5^ Frazer Institute, Faculty of Medicine, The University of Queensland, Brisbane, Queensland 4102, Australia.

^6^ Department of Anatomy and Developmental Biology, Monash University, Wellington Road, Clayton, VIC 3800, Australia

^7^ Computational Biology, Isomorphic Labs, Bishopsgate, London, England, EC2M 4RB, United Kingdom

* whom correspondence should be addressed. Email: ning.liu@adelaide.edu.au

**This supplementary information file includes:**

Supplementary Figures:

- Supplementary figure 1 to 19

Supplementary figure 1. The impact of different parameter k makes in the calculated probabilities. Different k (10, 50, 100, 200, 1000) were used in hoodscanR for identifying neighborhood for cells from the 10X Xenium breast cancer dataset. Resulting probability matrices were used to calculated pairwise Pearson correlation.

Supplementary figure 2. The impact of different parameter tau makes in the calculated probabilities. X axis is the 100 nearest cells of any cells in the 10X Xenium breast cancer dataset. Y axis is the accumulated probabilities calculated for the nearest cells. Noted that tau = 503.894 here is the default tau for the hoodscanR package.

Supplementary figure 3. Maps of pathological annotation and detected spatial domains by different methods with 10X Xenium breast cancer data.

Supplementary figure 4. Maps of pathological annotation and detected spatial domains by different methods with 10X Xenium breast cancer data.

Supplementary figure 5. Maps of pathological annotation and detected spatial domains by different methods with Nanostring CosMx NSCLC data.

Supplementary figure 6. Maps of known domains and detected spatial domains by different methods with MERFISH mouse colon data. Domains include muscularis externa (ME), mucosa (MU), submucosa (SM) and other.

Supplementary figure 7. Maps of known domains and detected spatial domains by different methods with STARmap mouse cortex data.

Supplementary figure 8. Testing robustness of hoodscanR with different level of resolution of annotation. A: Spatial map of 10X Xenium breast cancer data that colored by cell types. From top to bottom is high resolution annotation with T cell subtypes, medium resolution annotation with T cell subtypes grouped into one T_cells group, and low resolution annotation with all immune cells merged into one Immune group. B: Neighborhood-based spatial domains identification results from different resolution of annotation, with calculated NMI in the sub-titles (high resolution annotation as reference).

Supplementary figure 9. The identification of neighborhood-based clusters for three consecutive tissue slides from Nanostring CosMx NSCLC data. Left panels are the spatial plot with colors denoting the identified neihborhood clusters. Right panels are box plots showing the neighborhood profile of each cluster.

Supplementary figure 10. VissE result of gene-sets enriched for up-regulated genes comparing tumor cells from macrophage and stromal neighborhood. Left panel are word cloud plots describing gene-set clusters of different biological themes, middle panel is the gene-set overlap network graph of gene-sets, and right panel is the fold-change (log2-scaled) for genes belonging to each gene-set cluster.
